# Influence of pre-stroke dependency on safety and efficacy of endovascular therapy: A systematic review and meta-analysis

**DOI:** 10.3389/fneur.2022.956958

**Published:** 2022-09-21

**Authors:** Hengxiao Zhao, Xuesong Bai, Wei Li, Qiuyue Tian, Wenjiao Wang, Xiaofan Guo, Yao Feng, Linyan Duan, Adam A. Dmytriw, Aman B. Patel, Tingyu Yi, Wenbo Cao, Xiaoli Min, Wenhuo Chen, Liqun Jiao

**Affiliations:** ^1^Department of Neurosurgery, Xuanwu Hospital, Capital Medical University, Beijing, China; ^2^China International Neuroscience Institute (China-INI), Beijing, China; ^3^Department of Neurosurgery, Liaocheng Brain Hospital, Liaocheng, China; ^4^Beijing Key Laboratory of Clinical Epidemiology, School of Public Health, Capital Medical University, Beijing, China; ^5^Department of Library, Xuanwu Hospital, Capital Medical University, Beijing, China; ^6^Department of Neurology, Loma Linda University Health, Loma Linda, CA, United States; ^7^Department of Radiology and Nuclear Medicine, Xuanwu Hospital, Capital Medical University, Beijing, China; ^8^Beijing Key Laboratory of Magnetic Resonance Imaging and Brain Informatics, Beijing, China; ^9^Neuroendovascular Program, Massachusetts General Hospital, Harvard Medical School, Boston, MA, United States; ^10^Department of Neurology, Zhangzhou Affiliated Hospital, Fujian Medical University, Fuzhou, China; ^11^Department of Cerebrovascular Diseases, The Second Affiliated Hospital, Kunming Medical University, Kunming, China; ^12^Department of Interventional Neuroradiology, Xuanwu Hospital, Capital Medical University, Beijing, China

**Keywords:** ischemic stroke, patient selection, treatment outcome, meta-analysis, systematic review, endovascular therapy (EVT)

## Abstract

**Background and purpose:**

In the landmark trials studying endovascular thrombectomy (EVT), pre-stroke dependent (PSD) patients were generally excluded. This systematic review and meta-analysis aimed to compare the safety and efficacy of EVT between PSD and pre-stroke independent (PSI) patients.

**Methods:**

We searched CENTRAL, Embase, and Ovid MEDLINE up to 11 November 2021 for studies assessing PSD and PSI patients, which were separately defined as pre-stroke mRS score >2 or >1, and ≤2 or ≤1 accordingly. Two authors extracted data and assessed the risk of bias. A meta-analysis was carried out using the random-effects model. Adjusted OR and 95% CI were used to estimate adjusted pool effects. The main outcomes included favorable outcomes, successful recanalization, symptomatic intracranial hemorrhage, and 90-day mortality.

**Results:**

A total of 8,004 records met the initial search strategy, and ten studies were included in the final decision. Compared with PSI_mRS≤2_, PSD_mRS>2_ had a lower favorable outcome (OR 0.51; 95% CI, 0.33–0.79) and higher 90-day mortality (OR 3.32; 95% CI, 2.77–3.98). No significant difference was found in successful recanalization and sICH. After adjustment, only 90-day mortality (aOR 1.99; 95% CI, 1.58–2.49) remained significantly higher in PSD_mRS>2_. Compared with PSI_mRS≤1_, PSD_mRS>1_ had lower 90-day mortality (OR, 3.10; 95% CI, 1.84–5.24). No significant difference was found regarding the favorable outcome, successful recanalization, and sICH. After adjustment, no significant difference was found in a favorable outcome, but a higher rate of 90-day mortality (aOR, 2.13; 95% CI, 1.66–2.72) remained in PSD_mRS>1_.

**Conclusions:**

PSD does not innately influence the EVT outcomes regarding sICH and favorable outcomes but may increase the risk of 90-day mortality. Until further evidence is available, it is reasonable to suggest EVT for patients with PSD.

## Introduction

Endovascular thrombectomy (EVT) has been proven to be an effective therapy for patients with acute ischemic stroke (1). However, most randomized controlled trials (RCTs) and real-world research limited investigation of patients with a pre-stroke modified Rankin scale (mRS) score of 0–1, and those with a score of ≥2 were largely excluded (2). There are studies showing that even with a higher mRS score, pre-stroke dependent (PSD) patients may also benefit from EVT when compared with best medical therapy (3, 4). It is less clear whether these patients achieve similar safety and efficacy as pre-stroke independent patients (PSI) after EVT. Recently, this issue has been hotly debated with inconsistent results among studies. A study conducted by Goda et al. indicated that PSD patients might have an extremely poor prognosis (5), while others indicated that PSD was not associated with less-favorable outcomes and should not be an exclusion criterion for EVT (6, 7). Therefore, in order to maximally expand the benefit of EVT in patients with acute ischemic stroke, it is necessary to clarify the impact of PSD on the outcome after EVT. This systematic review and meta-analysis aimed to provide physicians and neurointerventionalists with updated and reliable evidence of patient selection when performing EVT.

## Methods

### Search strategy

We searched the Cochrane Central Register of Controlled Trails (CENTRAL), Embase, Ovid MEDLINE, and EPub ahead of the print, in-process, in-data review, and other non-indexed citations for relevant studies published from 1946 to 11 November 2021. We used the key terms *endovascular procedures, vascular surgical procedures, thrombectomy, embolectomy, stents and cerebrovascular disorders, basal ganglia cerebrovascular disease, brain ischemia, carotid artery diseases, carotid artery thrombosis, intracranial arterial diseases, cerebral arterial diseases, intracranial embolism and thrombosis, and stroke* as keywords. The search strategy table was listed in the Supplementary material (see Supplementary File 1 Search strategy). The study was not pre-registered.

### Study selection

Two independent reviewers (YF and XG) searched the aforementioned main databases for study selection. EndNote software (version 20) was used to manage and search for studies. First, reviewers searched for titles and abstracts to exclude irrelevant articles. Subsequently, full articles were obtained and assessed for inclusion. The reasons for inclusion or exclusion were recorded in detail. When encountering any discrepancy between the two reviewers, a third reviewer (XB) was consulted to adjudicate.

### Study criteria

The inclusion criteria were RCTs or observational studies of adult patients with ischemic stroke managed with EVT. Studies were excluded if they failed to provide a PSI group.

### Patient selection criteria

Patients aged ≥18 who received EVT due to ischemic stroke were included. PSD was separately defined as a pre-stroke mRS score of >2 or >1, and PSI was defined as a pre-stroke mRS score of ≤2 or ≤1 accordingly. Patients were excluded if their mRS score was missing.

### Outcome

1. Favorable outcomes, defined as an mRS score of 0–2 or no greater than the pre-stroke mRS score.
2. Successful recanalization, defined as a modified Thrombolysis in Cerebral Infarction scale (m-TICI) 2b-3.
3. Symptomatic intracranial hemorrhage (sICH), defined as intracranial hemorrhage on imaging and ≥ 4 points increase on the National Institutes of Health Stroke Scale (NIHSS) within 24-h post-intervention in accordance with the second European Australasian Acute Stroke Study classification.
4. 90-day mortality.

### Assessment of risk bias and heterogeneity

Two reviewers (YF and XG) independently assessed the risk of bias and heterogeneity. The Cochrane Collaboration criteria were used for RCTs, and the Newcastle-Ottawa Scale was used for observational studies in the process of risk of bias assessment (8). Selection bias, performance bias, detection bias, attrition bias, and reporting bias of the selected studies were taken into consideration. Heterogeneity was evaluated by *I*^2^, where >50% represented substantial heterogeneity. Der Simonian and Laird's random-effects model was used for pooling outcomes. Sensitivity analysis was also used to find out the reasons for heterogeneity.

### Data extraction and statistical analysis

Two reviewers (YF and XG) independently collected data from studies under a predefined standard. The information included is as follows: (1) study characteristics—authors, publication time, country of the involved patients, type of studies, and Newcastle-Ottawa Scale score were included in the first part. (2) Patient characteristics—demographic characteristics included the number of PSD and PSI patients, gender, and age. Vascular risk factors included hypertension, diabetes mellitus, coronary artery disease, peripheral artery disease, smoking, and dyslipidemia. Medical history included myocardial infarction, atrial fibrillation, and previous transient ischemic attack (TIA) or stroke. Anti-thrombotic drugs taken prior to a stroke included anticoagulants and antiplatelets. The location of the occlusion, onset characteristics, etiology of stroke, and intervention characteristics were also recorded in detail. (3) Outcomes were as aforementioned, including favorable outcome, successful recanalization, sICH, and 90-day mortality. We tried to contact the corresponding authors of the study for any missing or wrong data.

A random-effects model was utilized by computing odds ratios (OR) and 95% confidential intervals (CI). In addition, adjusted OR and 95% CI were used to estimate adjusted pool effects. Publication bias was assessed by means of a funnel plot and Egger's test. Statistical analyses were performed using STATA version 15.0 (StataCorp LP, College Station, TX, USA).

## Results

### Study selection and study characteristics

**Figure 1:**
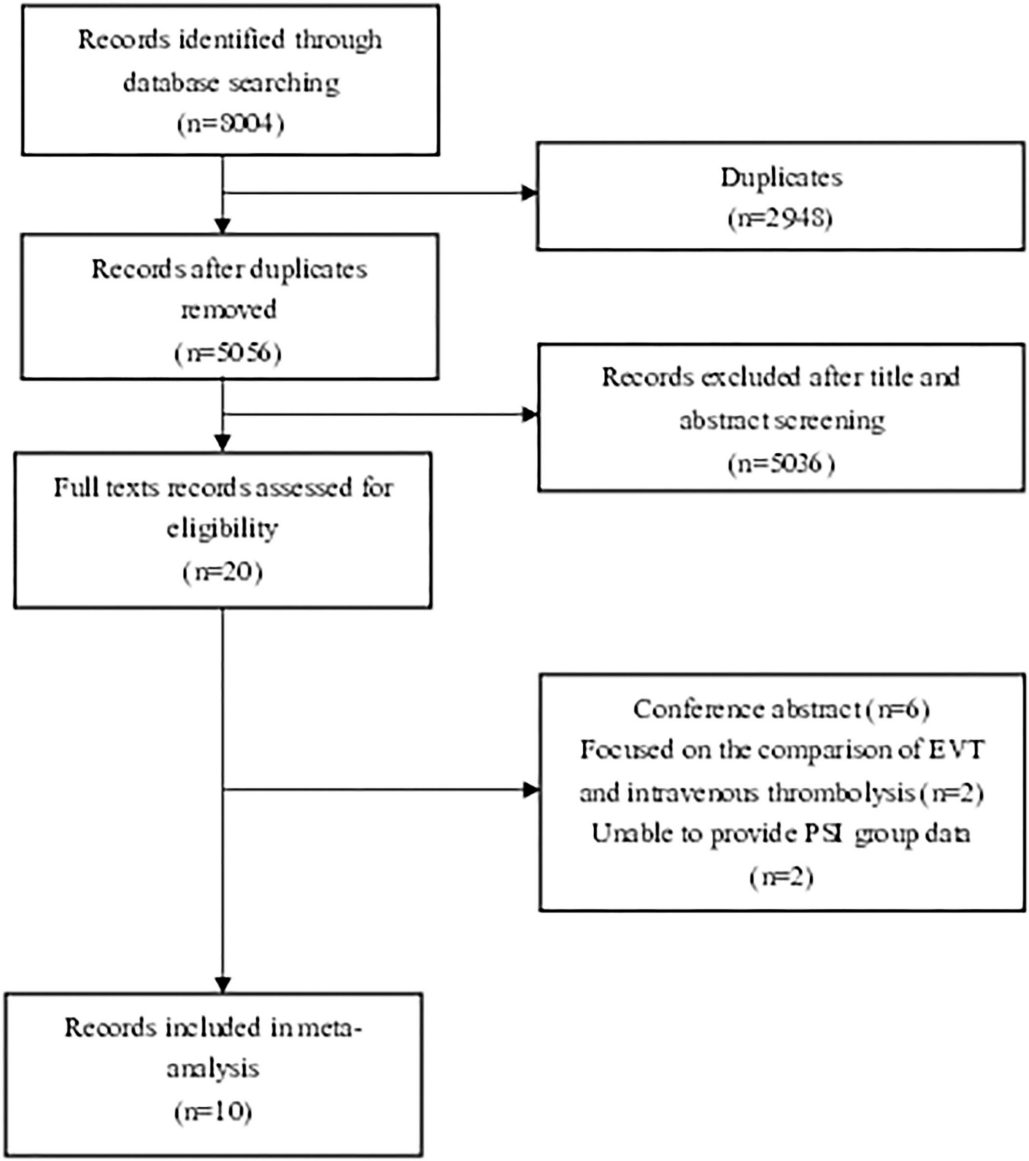
Flow chart illustrating study selection.

We found 8,004 records that met the initial standard after checking the main database at the first search step. Twenty articles were initially identified by title and abstract. Articles that did not meet the study criteria and type were excluded. Six conference abstracts were excluded (9–14). Two articles focusing on the comparison of PSD patients undergoing EVT vs. standard medical treatment (SMT) were excluded (3, 4). Two articles that failed to compare PSD and PSI groups were also excluded (15, 16). Finally, six articles which defined PSD as pre-stroke mRS > 2 (6, 7, 17–20) and four articles which defined PSD as pre-stroke mRS score >1 (5, 21–23) were eligible for meta-analysis after reading full texts. The flow diagram is shown below to demonstrate the procedure of study selection (Figure 1).

As shown in Tables 1, 2, a total of 10 studies and 8,823 patients met the inclusion criteria. All studies included patients with anterior circulation stroke, except one study with a small complement of posterior circulation stroke cases (18). All studies were published after 2017, with five conducted in Europe (6, 17, 18, 22), two in Asia (5, 7), 2 in North America (21, 23), and 1 in Oceanica (19). Five studies were multicenter trails (6, 19, 21–23) and five were single-center studies (5, 7, 17, 18, 20).

**Table 1:** Baseline characteristics of the PSD_mRS>2_ group.

	**Florent et al**. **(****17****)**		**Goldhoorn et al**. **(****6****)**		**Larsson et al**. **(****18****)**		**Nababan et al**. **(****19****)**		**Oesch et al**. **(****20****)**		**Leker et al**. **(****7****)**	
**Basic information**													
Publication time	2021		2018		2020		2021		2021		2017	
Country	France		Netherland		Sweden		Australia		Switzerland		Israel	
Type of studies	Single center		Multicenter		Single center		Multicenter		Single center		Single center	
NOS score	7		6		7		5		6		6	
**Demographic characteristics**													
	PSD (***n =*** 155)	PSI (***n =*** 767)	PSD (***n =*** 157)	PSI (***n =*** 1284)	PSD (***n =*** 90)	PSI (***n =*** 501)	PSD (***n =*** 82)	PSI (***n =*** 720)	PSD (***n =*** 84)	PSI (***n =*** 1163)	PSD (***n =*** 23)	PSI (***n =*** 108)
Gender, male, ***n*** (%)	40 (25.8)	383 (49.9)	64 (41)	706 (55)	36 (40.0)	283 (56.5)	27 (33)	395 (55)	32 (38.1)	628 (54.0)	9 (39)	56 (52)
Age (years) Mean ± SD Median [IQR]	80.3 ± 12.4	67.4 ± 14.8	80 (71–86)	69 (59–78)	86 (57)	74 (82)	85 (78–89)	73 (62–82)	81 (73.75–85)	72 (60–79)	80.3 ± 10	66.9 ± 14
**Vascular risk factors**													
Hypertension, ***n*** (%)	125 (80.6)	481 (62.7)	97 (62)	618 (48)	65 (73.9)	322 (64.9)	NR	NR	67 (79.8)	784 (67.6)	20 (87)	79 (73)
Diabetes mellitus, ***n*** (%)	36 (23.2)	144 (18.8)	46 (29)	197 (15)	20 (22.2)	84 (16.8)	NR	NR	26 (31.0)	185 (15.9)	8 (35)	38 (35)
Coronary artery disease, ***n*** (%)	NR	NR	NR	NR	NR	NR	NR	NR	14 (17.1)	217 (18.8)	NR	NR
Peripheral artery disease, ***n*** (%)	NR	NR	23 (15)	109 (8)	NR	NR	NR	NR	NR	NR	NR	NR
Smoker, ***n*** (%)	40 (25.8)	311 (40.5)	30 (19)	297 (23)	NR	NR	NR	NR	9 (14.1)	237 (22.3)	4 (17)	33 (31)
Dyslipidemia, ***n*** (%)	65 (41.9)	338 (44.1)	59 (38)	351 (27)	30 (34.1)	197 (40.5)	NR	NR	43 (52.4)	661 (57.6)	14 (61)	51 (47)
**Medical history**													
Myocardial infarction, ***n*** (%)	21 (13.5)	90 (11.7)	45 (29)	170 (13)	NR	NR	NR	NR	NR	NR	15 (26)	43 (40)
Atrial fibrillation, ***n*** (%)	102 (65.8)	269 (35.1)	58 (37)	262 (20)	56 (62.2)	204 (40.9)	NR	NR	NR	NR	14 (61)	56 (52)
Previous TIA or stroke, ***n*** (%)	50 (32.3)	130 (16.9)	52 (33)	183 (14)	25 (90)	66 (13.2)	NR	NR	27 (32.9)	105 (9.1)	9 (39)	18 (17)
**Anti-thrombotic drugs prior to stroke**													
Anticoagulants, ***n*** (%)	50 (32.3)	122 (15.9)	40 (25)	145 (11)	NR	NR	NR	NR	54 (65.1)	491 (42.6)	NR	NR
Antiplatelets, ***n*** (%)	70 (45.2)	218 (28.4)	70 (45)	400 (31)	NR	NR	NR	NR	NR	NR	NR	NR
**Loction of the occlusion**													
M1-MCA, ***n*** (%)	97 (63.0)	363 (47.5)	98 (62)	704 (55)	NR	NR	48 (59)	385 (53)	NR	NR	15 (65)	73 (68)
M2-MCA, ***n*** (%)	23 (14.9)	113 (14.8)	15 (10)	154 (12)	NR	NR	18 (22)	177 (25)	NR	NR	NR	NR
ICA+MCA, ***n*** (%)	30 (19.4)	278 (36.2)	NR	NR	NR	NR	NR	NR	NR	NR	5 (22)	13 (12)
Isolated M1 MCA, ***n*** (%)	NR	NR	NR	NR	NR	NR	NR	NR	NR	NR	2 (9)	11 (10)
Isolated ICA occlusion, ***n*** (%)	4 (2.6)	11 (1.4)	29 (18)	273 (21)	NR	NR	NR	NR	NR	NR	NR	NR
ICA, ***n*** (%)	NR	NR	5 (3)	75 (6)	30 (33.3)	152 (30.3)	16 (20)	153 (21)	NR	NR	1 (4)	11 (10)
MCA, ***n*** (%)	NR	NR	NR	NR	43 (47.8)	275 (54.8)	NR	NR	NR	NR	NR	NR
Other: M3/anterior/vertebral/ basilar/posterior, ***n*** (%)	NR	NR	5 (3)	11 (1)	12 (13.3)	97 (19.4)	0 (0)	5 (1)	NR	NR	NR	NR
Left hemisphere, ***n*** (%)	NR	NR	91 (58)	683 (53)	NR	NR	NR	NR	NR	NR	17 (74)	57 (53)
Systolic blood pressure, mean mm Hg (SD)	NR	NR	149 (26)	150 (24)	NR	NR	NR	NR	NR	NR	NR	NR
Initial median NIHSS score Mean ± SD, Median [IQR]	20 (16–23)	17 (13–21)	17 (13–20)	16 (11–20)	18 (9)	16 (8)	17 (12–20)	15 (9–19)	18 (11–21)	15 (10–19)	19.7 ± 5.9	16.0 ± 6.6
ASPECTS Mean ± SD, Median [IQR]	8 (6–9)	7 (5–9)	9 (8–10)	9 (7–10)	NR	NR	9 (8–10)	9 (7–10)	NR	NR	NR	NR
**Causes of stroke**													
Large artery atherosclerosis, ***n*** (%)	18 (11.6)	118 (15.4)	NR	NR	NR	NR	NR	NR	5 (6)	170 (14.6)	NR	NR
Cardioembolic, ***n*** (%)	100 (64.5)	347 (45.2)	NR	NR	NR	NR	NR	NR	41 (48.8)	485 (41.7)	15 (65)	73 (67)
Other, ***n*** (%)	5 (3.2)	44 (5.7)	NR	NR	NR	NR	NR	NR	3 (3.6)	70 (6.0)	NR	NR
Unknown, ***n*** (%)	32 (20.6)	258 (33.6)	NR	NR	NR	NR	NR	NR	35 (41.7)	438 (37.7)	NR	NR
**Intervention characteristics**													
General anesthesia, ***n*** (%)	2 (1.3)	14 (1.8)	NR	NR	NR	NR	NR	NR	NR	NR	NR	NR
IVT, ***n*** (%)	75 (48.4)	534 (69.6)	100 (64)	1023 (80)	38 (42.2)	252 (50.4)	11 (13)	111 (15)	NR	NR	10 (45)	36 (33)
Duration of intervention (min), Mean ± SD, Median [IQR]	NR	NR	61 (45–85)	64 (40–90)	NR	NR	34 (23–50)	30 (20–47)	NR	NR	NR	NR
Onset to recanalization, Mean ± SD, Median [IQR]	NR	NR	291 (222–338)	266 (216–330)	NR	NR	245 (170–363)	273 (180–521)	NR	NR	254 ± 126	288 ± 176

PSD_**mRS>2**_, pre-stroke dependent, was defined as modified Rankin scale score > 2; NOS, Newcastle-Ottawa Scale; SD, standard deviation; IQR, interquartile range; TIA, transient ischemic attack; MCA, middle cerebral artery; M1, a segment of the MCA; M2, a segment of the MCA; M3, a segment of the MCA; ICA, intracranial carotid artery; NIHSS, National Institutes of Health Stroke Scale; ASPECTS, Alberta Stroke Program Early CT Score; IVT, intravenous thrombolysis; NR, not reported.

**Table 2:** Baseline characteristics of the PSD_**mRS>1**_ group.

	**Havenon et al**. **(****21****)**		**Millan et al**. **(****22****)**		**Salwi et al**. **(****23****)**	
**Basic information**						
Publication time	2021		2021		2020	
Country	United States		Spain		United States	
Type of studies	Multicenter		Multicenter		Multicenter	
NOS score	6		6		6	
**Demographic characteristics**						
	PSD (*n =* 53)	PSI (*n =* 354)	PSD (*n =* 409)	PSI (*n =* 1978)	PSD (*n =* 259)	PSI (*n =* 502)
Gender, male, *n* (%)	18 (34.0)	191 (54.0)	171 (42)	1031 (52)	96 (37.1)	268 (53.4)
Age (years) Mean ± SD Median [IQR]	73.3 ± 16.5	65.2 ± 14.5	77 ± 11	70 ± 13	80 (67–88)	67 (57–77)
**Vascular risk factors**						
Hypertension, *n* (%)	41 (77.4)	262 (74.0)	279 (71)	1026 (55)	221 (85.3)	348 (69.3)
Diabetes mellitus, *n* (%)	11 (20.8)	91 (25.7)	86 (22)	313 (17)	72 (27.8)	112 (22.3)
Smoker, *n* (%)	9 (17.0)	81 (23.0)	29 (7)	281 (15)	NR	NR
Dyslipidemia, *n* (%)	29 (54.7)	171 (48.3)	177 (45)	731 (39)	NR	NR
**Medical history**						
Atrial fibrillation, *n* (%)	37 (69.8)	142 (40.1)	151 (38)	413 (22)	147 (56.8)	170 (33.9)
Previous TIA or stroke, *n* (%)	NR	NR	60 (15)	153 (8)	NR	NR
**Anti-thrombotic drugs prior to stroke**						
Anticoagulants, *n* (%)	NR	NR	97 (25)	261 (14)	49 (18.9)	63 (12.5)
Antiplatelets, *n* (%)	NR	NR	NR	NR	123 (47.5)	187 (37.3)
**Loction of the occlusion**						
M1-MCA, *n* (%)	NR	NR	198 (48)	973 (49)	161 (62.2)	310 (61.8)
M2-MCA, *n* (%)	NR	NR	80 (19)	354 (18)	47 (18.1)	84 (16.7)
Isolated ICA occlusion, *n* (%)	NR	NR	57 (14)	277 (14)	NR	NR
ICA, *n* (%)	NR	NR	32 (8)	110 (6)	48 (18.5)	106 (21.1)
Other: M3/anterior/vertebral/basilar/tandem, *n* (%)	NR	NR	264 (13)	43 (11)	3 (1.2)	2 (0.4)
Left hemisphere, *n* (%)	NR	NR	218 (53)	1097 (56)	NR	NR
Initial median NIHSS score Mean ± SD, Median [IQR]	18 (13-24)	17 (13-21)	17 (12–20)	17 (11–21)	17 (12–22)	15 (10–20)
ASPECTS Mean ± SD, Median [IQR]	NR	NR	10 (8–10)	9 (8–10)	9 (8–10)	9 (8–10)
**Intervention characteristics**	NR	NR	NR	NR	NR	NR
IVT, *n* (%)	18 (34.0)	199 (56.2)	139 (34)	835 (42)	113 (43.6)	263 (52.4)
Onset to recanalization, Mean ± SD, Median [IQR]	NR	NR	281 (203–431)	300 (210–466)	273.5 (190.0–431.5)	289.5 (198.75–458.5)
Onset to puncture, Mean ± SD, Median [IQR]	228.5 (183-312)	283 (193-430)	NR	NR	NR	NR

PSD_mRS>1_, pre-stroke dependent, was defined as modified Rankin scale score > 1; NOS, Newcastle-Ottawa Scale; SD, standard deviation; IQR, interquartile range; TIA, transient ischemic attack; MCA, middle cerebral artery; M1, a segment of the MCA; M2, a segment of the MCA; ICA, intracranial carotid artery; NIHSS, National Institutes of Health Stroke Scale; ASPECTS, Alberta Stroke Program Early CT Score; IVT, intravenous thrombolysis; NR, not reported.

Among the six studies defining PSD as pre-stroke mRS score >2, a total of 591 patients were identified in the PSD_mRS>2_ group and 4,543 patients in the PSI_mRS≤2_ group. The majority of PSD_mRS>2_ patients were females (383, 64.8%), but most PSI_mRS≤2_ patients were males (2451, 54.0%). The mean NIHSS score ranged from 17 to 20 in the PSD_mRS>2_ group and 15 to 17 in PSI_mRS≤2_ group, respectively. Hypertension was the most common vascular risk factor (PSD_mRS>2_, 73.8%; PSI_mRS≤2_, 60.0%). The majority of occlusions were of the middle cerebral artery (MCA) (PSD_mRS>2_, 77.9%; PSI_mRS≤2_, 88.5%; total, 86.9%), and the M1 segment was the most common in MCA occlusion (PSD_mRS>2_, 66.0%; PSI_mRS≤2_, 60.3%; total, 61.1%).

Among the recruited four studies defining PSD as pre-stroke mRS score >1, one study based its baseline characteristics on patients with different 90-day mRS scores, so we were unable to extract baseline data for PSD and PSI groups separately (5). As a result, the Goda et al. (5) Japanese single-center trail (PSD, *n* = 51; PSI, *n* = 83; NOS score, 6) was ultimately ineligible for data extraction of baseline characteristics. A total of 721 patients were classified as PSD_mRS>1_ and 2,834 were classified as PSI_mRS≤1_ of 3 studies. The majority of PSD_mRS>1_ patients were females (436, 60.5%), but a higher proportion of PSI_mRS≤1_ patients were males (1490, 52.6%). The mean NIHSS score ranged from 12 to 24 in the PSD_mRS>1_ group and 10 to 21 in PSI_≤1_ group, respectively. Hypertension was the most common vascular risk factor (PSD_mRS>1_, 76.7%; PSI_mRS≤1_, 60.1%). The most common site of occlusion was the MCA (PSD_mRS>1_, 72.8%; PSI_mRS≤1_, 69.4%; total, 70.1%), and the M1 segment was the most common in that segment (PSD_mRS>1_, 73.9%; PSI_mRS≤1_, 74.5%; total, 74.4%).

### Risk of bias

We used the Newcastle-Ottawa Scale to assess the quality of the studies and the risk of bias (see Supplementary File 2). All studies were of high quality, which implies a low risk of bias. Funnel plots were used to assess the risk of publication bias (Supplementary Files 3–6, 11–13) When comparing the PSD_mRS>2_ group with the PSI_mRS≤2_ group, the Egger test *p*-value for a favorable outcome, successful recanalization, sICH, and 90-day mortality were 0.967, 0.893, 0.895, and 0.976, which suggests no evidence of publication bias (see Supplementary Files 7–10). After adjustment, the Egger test *p-*value for favorable outcomes and sICH, 90-day mortality were 0.515, 0.535, and 0.448, suggesting no evidence of publication bias (see Supplementary Files 14–16). When comparing PSD_mRS>1_ group with PSI_mRS≤1_ group, the Egger test *p-*value for a favorable outcome and 90-day mortality were 0.964 and 0.431, which suggests no evidence of publication bias (see Supplementary Files 19, 20). After adjustment, the Egger test *p-*value for a favorable outcome and 90-day mortality were 0.522 and 0.376, respectively, which also suggests no evidence of publication bias (see Supplementary Files 23, 24).

### Meta-analysis of outcomes

**Figure 2:**
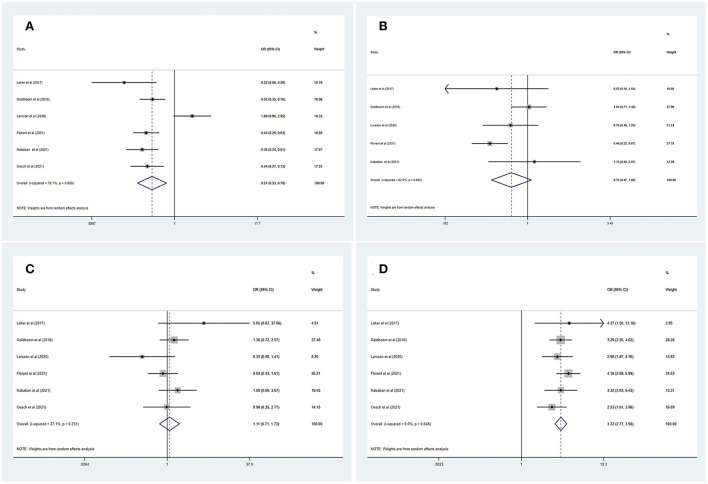
Forest plots of meta-analyses of PSD_mRS>2_ primary outcomes based on OR. **(A)** Favorable outcome; **(B)** Successful recanalization; **(C)** Symptomatic intracranial hemorrhage; and **(D)** 90-day mortality.

**Figure 3:**
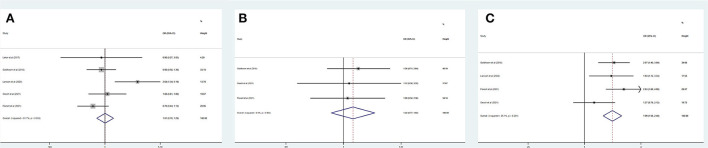
Forest plots of meta-analyses of PSD_mRS>2_ primary outcomes based on aOR. **(A)** Favorable outcome; **(B)** Symptomatic intracranial hemorrhage; and **(C)** 90-day mortality.

Table 3 summarizes the main outcomes of the meta-analysis in PSD_mRS>2_ and PSI_mRS≤2_ patients. The rate of a favorable outcome in PSD_mRS>2_ and PSI_mRS≤2_ patients was 27.8 and 43.7%, respectively. There were 71.3% of PSD_mRS>2_ patients and 76.5% of PSI_mRS≤2_ who achieved successful recanalization. The rate of sICH was 6.6% in PSD_mRS>2_ patients and 6.1% in PSI_mRS≤2_ patients, respectively. The 90-day mortality rate was 46.9% in PSD_mRS>2_ patients and 21.8% in PSI_mRS≤2_ patients. Using a random-effects model, pooling of six studies showed a significant difference in favorable outcomes (OR, 0.51; 95% CI, 0.33–0.79; *p* = 0.000; *I*^2^ = 78.1%) (Figure 2A) and 90-day mortality (OR, 3.32; 95% CI, 2.77–3.98; *p* = 0.648; *I*^2^ = 0.0%) (Figure 2D) between the PSD_mRS>2_ and PSI_mRS≤2_ groups. There was no significant difference in successful recanalization (OR, 0.72; 95% CI, 0.47–1.08; *p* = 0.032; *I*^2^ = 62.0%) (Figure 2B) and sICH (OR, 1.11; 95% CI, 0.71-1.73; *p* = 0.231; *I*^2^ = 27.1%) (Figure 2C) between the two groups. Considering potential confounding bias from baseline differences between the two groups of patients, we further extracted aOR from the original articles to investigate the results mentioned above. There was no significant difference in favorable outcome (aOR, 1.01; 95% CI, 0.78–1.29; *p* = 0.033; *I*^2^ = 67.1%) (Figure 3A) and sICH (aOR, 1.22; 95% CI, 0.77–1.92; *p* = 0.902; *I*^2^ = 0.0%) (Figure 3B) between the PSD_mRS>2_ and PSI_mRS≤2_ groups after adjustment. A significant difference remained in 90-day mortality (aOR, 1.99; 95% CI, 1.58–2.49; *p* = 0.201; *I*^2^ = 35.1%) (Figure 3C). For successful recanalization outcome, we could not estimate pooled effect because only one study reported an aOR (17).

**Table 3:** The PSD_mRS>2_ group's analysis of outcomes.

**Outcomes**	**OR (95% CI)**	**Lower**	**Upper**	***I*^2^(%)**	***p***	**aOR (95% Cl)**	**Lower**	**Upper**	***I*^2^(%)**	***p***
Favorable outcome	0.51	0.33	0.79	78.1	0.000	1.01	0.78	1.29	67.1	0.033
Successful recanalization	0.72	0.47	1.08	62.0	0.032	NR	NR	NR	NR	NR
Symptomatic ICH	1.11	0.71	1.73	27.1	0.231	1.22	0.77	1.92	0.0	0.902
90 day-mortality	3.32	2.77	3.98	0.0	0.648	1.99	1.58	2.49	35.1	0.201

PSD_mRS>2_, pre-stroke dependent, was defined as modified Rankin scale score > 2; OR, odds ratio; CI, confidence interval; *I*^2^, the variation attributable to heterogeneity; aOR, adjusted odds ratio; ICH, intracranial hemorrhage; NR, not reported.

**Figure 4:**
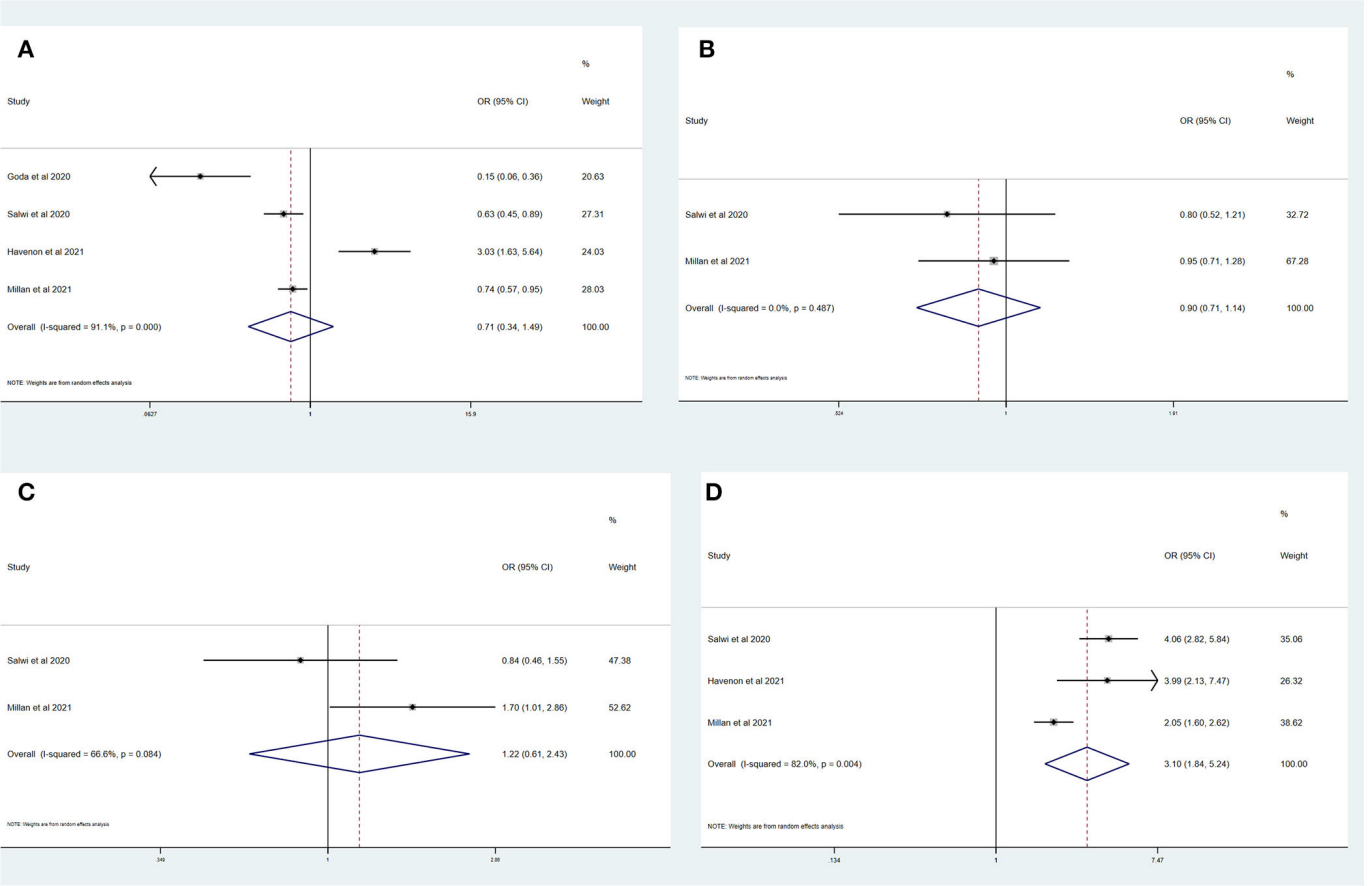
Forest plots of meta-analyses of PSD_mRS>1_ primary outcomes based on OR. **(A)** Favorable outcome; **(B)** Successful recanalization; **(C)** Symptomatic intracranial hemorrhage; and **(D)** 90-day mortality.

**Figure 5:**
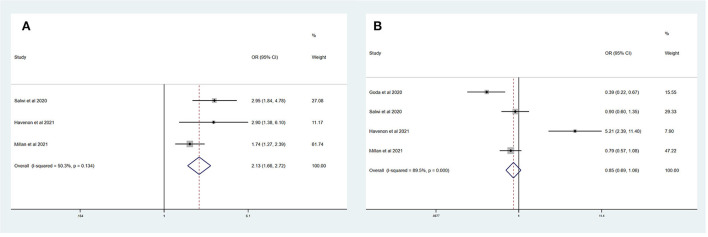
Forest plots of meta-analyses of PSD_mRS>1_ primary outcomes based on aOR. **(A)** 90-day mortality and **(B)** Favorable outcome.

Table 4 summarizes the main outcomes of a meta-analysis in PSD_mRS>1_ and PSI_mRS≤1_ patients. The rate of a favorable outcome in PSD_mRS>1_ and PSI_mRS≤1_ patients was 25.3 and 30.2%, respectively. In total, 84.0% of PSD_mRS>1_ patients and 85.1% of PSI_mRS≤1_ patients achieved successful recanalization. The rate of sICH was 5.5% in PSD_mRS>1_ patients and 3.9% in PSI_mRS≤1_ patients, respectively. The 90-day mortality rate was 35.0% in PSD_mRS>1_ patients and 16.8% in PSI_mRS≤1_ patients. There remained a significant difference in 90-day mortality (OR, 3.10; 95% CI, 1.84–5.24; *p* = 0.004; *I*^2^ = 82.0%) (Figure 4D) between the PSD_mRS>1_ and PSI_mRS≤1_ groups. There was no significant difference in favorable outcome (OR, 0.71; 95% CI, 0.34–1.49; *p* = 0.000; *I*^2^ = 91.1%) (Figure 4A), successful recanalization (OR, 0.90; 95% CI, 0.71–1.14; *p* = 0.487; *I*^2^ = 0.0%) (Figure 4B) and sICH (OR, 1.22; 95% CI, 0.61–2.43; *p* = 0.084; *I*^2^ = 66.6%) (Figure 4C) between two groups. After adjustment, there was no significant difference in favorable outcome (aOR, 0.85; 95% CI, 0.69–1.06; *p* = 0.000; *I*^2^ = 89.5%) (Figure 5B) between the PSD_mRS>1_ and PSI_mRS≤1_ groups. A significant difference remained in 90-day mortality (aOR, 2.13; 95% CI, 1.66–2.72; *p* = 0.134; *I*^2^ = 50.3%) (Figure 5A). For successful recanalization outcome and sICH, we could not estimate the pooled effect as no study reported aOR for successful recanalization and only one study reported an aOR for sICH.

**Table 4:** The PSD_mRS>1_ group's analysis of outcomes.

**Outcomes**	**OR (95% CI)**	**Lower**	**Upper**	***I*^2^(%)**	***p***	**aOR (95% Cl)**	**Lower**	**Upper**	***I*^2^(%)**	***p***
Favorable outcome	0.71	0.34	1.49	91.1	0.000	0.85	0.69	1.06	89.5	0.000
Successful recanalization	0.90	0.71	1.14	0.0	0.487	NR	NR	NR	NR	NR
Symptomatic ICH	1.22	0.61	2.43	66.6	0.084	NR	NR	NR	NR	NR
90 day-mortality	3.10	1.84	5.24	82.0	0.004	2.13	1.66	2.72	50.3	0.134

PSD_mRS>1_, pre-stroke dependent, was defined as modified Rankin scale score > 1; OR, odds ratio; CI, confidence interval; *I*^2^, the variation attributable to heterogeneity; aOR, adjusted odds ratio; ICH, intracranial hemorrhage; NR, not reported.

## Discussion

This systematic review and meta-analysis are the first to compare the safety and efficacy of EVT between PSD and PSI patients. The results of this study expand the existing knowledge regarding the efficacy of EVT in PSD patients who sustained AIS. Compared with the PSI_mRS≤2_ group, the PSD_mRS>2_ group had a lower rate of favorable outcome (OR, 0.51; 95% CI, 0.33–0.79) and a higher rate of 90-day mortality (OR, 3.32; 95% CI, 2.77–3.98). Both groups had a comparable rate of successful recanalization and sICH. After adjustment, only 90-day mortality was still significantly higher in the PSD_mRS>2_ group (aOR, 1.99; 95% CI, 1.58–2.49). Compared with the PSI_mRS≤1_ group, PSD_mRS>1_ group had a higher rate of 90-day mortality (OR, 3.10; 95% CI, 1.84–5.24). No significant difference was found in favorable outcomes, successful recanalization, and sICH. After adjustment, a higher rate of 90-day mortality (aOR, 2.13; 95% CI, 1.66–2.72) remained in the PSD_mRS>1_ group. Thus, in both the PSD_mRS>2_ group and PSD_mRS>1_ group, PSD patients were found to achieve a comparably favorable outcome, but there is an expectedly higher mortality risk for PSD compared to PSI patients.

Several causes may contribute to PSD status, such as dementia, cognitive impairment, and other medical comorbidities (7), but these were not specifically documented in most studies. It should be noted that previous RCTs have also generally excluded this group of patients (24, 25). In addition, there are studies showing that PSD increases the mortality rate. It is less likely to achieve favorable outcomes after intravenous thrombolysis (26). With the population worldwide growing substantially older, clinicians are more likely to encounter the question of deciding whether to perform EVT on patients with neurological and non-neurological disabilities. Thus, clarifying the efficacy of EVT in PSD patients is crucial to meeting the evidence threshold to make challenging “real-world” decisions around the candidacy.

Pre-stroke dependent patients may have unique baseline characteristics, such as generally being older and harboring other comorbidities, especially cerebrovascular risk factors (20). This is also suggested by our compiled data showing hypertension was more likely to be present in PSD patients. Nevertheless, according to our results, performing EVT still seems effective in one-third of PSD patients, maintaining the same level of disability without progressing to a more debilitating state at 90-day follow-up. In some studies, a numerically lower rate of favorable outcomes was observed in PSD patients, and other variables may influence functional outcomes, such as age and stroke severity (17, 20). The compiled aORs in our study may support the notion that PSD is not the primary cause of the lower rate of favorable outcomes. Furthermore, sICH after EVT is closely associated with functional outcomes and has been shown to be comparable between both groups. Thus, the safety profile of EVT in PSD patients may be acceptable. It seems reasonable that large vessel occlusion stroke patients harboring pre-stroke disability should not be universally excluded from EVT.

Although maintaining a pre-stroke level of function after receiving EVT was 27.8% in the PSD_mRS>2_ group and 25.3% in the PSD_mRS>1_ group, respectively, higher mortality rates of 46.9% and 35.0% should not be overlooked. In the study by Larsson et al. (18) besides pre-stroke disability, other variables, including higher age, unsuccessful recanalization, comorbidities, and stroke severity, were risk factors for 90-day mortality. Fragility related to old age and comorbidity may be the primary reasons for the higher mortality in PSD patients. Thus, careful selection of PSD patients for EVT is necessary, and future studies with cost-effectiveness analysis and other detailed measurements are needed. At the same time, rates of successful reperfusion remain uncertain. In the study of Florent et al. (17) PSD patients were less likely to achieve successful reperfusion, and they ascribed it to several reasons, such as vascular anatomy, intravenous thrombolysis (IVT), anesthesia, and operator experience. More data are unmistakably needed to verify these suspicions.

## Limitations

There are several limitations inherent to this systematic review and meta-analysis. Studies recruited were not RCTs and could harbor unavoidable bias from the study design itself. We were unable to identify the primary cause of pre-stroke independence. Additionally, most of the studies were from European countries, and subgroup analysis based on different countries was impossible. Despite these shortcomings, this first relevant systematic review and meta-analysis provided the most updated clinical evidence of performing EVT in PSD patients. Future studies should investigate whether PSD patients with acute stroke might benefit more from EVT than SMT (e.g., IVT), but it is currently not available due to limited comparative studies (3, 4). Additionally, cost-effectiveness analyses and more detailed measurements of outcomes are required to better assess the true benefit and risk profiles when performing EVT in this particular group of patients.

## Conclusion

Pre-stroke dependent patients do not innately influence the EVT outcomes regarding sICH and favorable outcomes but may be associated with a higher risk of 90-day mortality. Thus, it seems reasonable to recommend EVT in selected PSD patients, although a greater amount of prospective evidence is needed.

## Data availability statement

The original contributions presented in the study are included in the article/Supplementary material, further inquiries can be directed to the corresponding author/s.

## Author contributions

XB and LJ developed the initial idea for this study. WW, YF, and LD developed and revised the search strategy. HZ, XB, XM, and LJ formulated the study design. WCh and LJ were consulted about clinical issues. HZ, XB, WL, and QT contributed to the original draft. HZ, XB, WL, XG, AD, AP, TY, WCb, XM, and LJ were responsible for revising the draft. All authors approved the final version of the manuscript before submission.

## Conflict of interest

The authors declare that the research was conducted in the absence of any commercial or financial relationships that could be construed as a potential conflict of interest.

## Publisher's note

All claims expressed in this article are solely those of the authors and do not necessarily represent those of their affiliated organizations, or those of the publisher, the editors and the reviewers. Any product that may be evaluated in this article, or claim that may be made by its manufacturer, is not guaranteed or endorsed by the publisher.
