# Supplementary material for: Influence of pre-stroke dependency on safety and efficacy of endovascular therapy: A systematic review and meta-analysis
Source: Front Neurol. 2022 Sep 21;13:956958. doi: 10.3389/fneur.2022.956958 (PMC9532553; doi:10.3389/fneur.2022.956958)
Supplement: Supplementary file 1 [file Data_Sheet_1.PDF]

Supplementary File 1 search strategy to 11 November 2021

Ovid MEDLINE(R) and Epub Ahead of Print, In-Process, In-Data-Review & Other Non-Indexed Citations, Daily and Versions(R) 1946 to November 10, 2021

#No.	Results	Searches
1	136509	endovascular procedures/ or vascular surgical procedures/ or exp thrombectomy/ or exp embolectomy/ or exp stents/
2	122044	(stent$ or thromboaspirat$ or thrombo-aspirat$ or thrombecto$ or embolecto$).tw.
3	7296	((clot or thrombus or thrombi or embol$) adj5 (aspirat$ or remov$ or retriev$ or retract$ or extract$)).tw.
4	34752	((mechanical or endovascular or neurovascular) adj5 (aspirat$ or treatment$ or Therap$)).tw.
5	81	((merci or concentric) adj5 retriever).tw.
6	206232	1 or 2 or 3 or 4 or 5
7	274665	cerebrovascular disorders/ or basal ganglia cerebrovascular disease/ or exp brain ischemia/ or carotid artery diseases/ or carotid artery thrombosis/ or intracranial arterial diseases/ or cerebral arterial diseases/ or exp "intracranial embolism and thrombosis"/ or exp stroke/
8	84009	(isch?emi$ adj5 (stroke$ or apoplex$ or cerebral vasc$ or cerebrovasc$ or cva or attack$)).tw.
9	123760	((brain or cerebr$ or cerebell$ or vertebrobasil$ or hemispher$ or intracran$ or intracerebral or infratentorial or supratentorial or middle cerebr$ or mca$ or anterior circulation) adj5 (isch?emi$ or infarct$ or thrombo$ or emboli$ or occlus$ or hypoxi$)).tw.
10	340356	7 or 8 or 9
11	22222	6 and 10
12	75357	exp Intracranial Hemorrhages/ or exp Basal Ganglia Hemorrhage/
13	66996	((brain$ or cerebr$ or cerebell$ or intracerebral or intracran$ or parenchymal or intraparenchymal or intraventricular or infratentorial or

		supratentorial or basal gangli$ or putaminal or putamen or posterior fossa or hemispher$) adj5 (h?emorrhag$ or h?ematoma$ or bleed$)).tw.
14	15559	((h?emorrhag$ or bleed$) adj5 (stroke or apoplex$)).tw.
15	13086	(ICH or ICHs).tw.
16	121661	12 or 13 or 14 or 15
17	3884	11 and 14
18	2795567	Epidemiologic studies/ or exp case control studies/ or exp cohort studies/ or Cross-sectional studies/
19	1657076	(Case control or (cohort adj (study or studies)) or Cohort analy$ or (Follow up adj (study or studies)) or (observational adj (study or studies)) or Longitudinal or Retrospective or Cross sectional).tw.
20	1278088	randomized controlled trial/ or clinical trial/ or controlled clinical trial/ or exp randomization/ or placebos/ or Control Groups/ or random allocation/ or double-blind method/ or single-blind method/ or Randomized Controlled Trials as Topic/ or exp Clinical Trials as Topic/
21	5417418	(randomized controlled trial or controlled clinical trial or clinical trial or multicenter study).pt. or randomized.ab. or placebo.ab. or drug therapy.fs. or randomly.ab. or trial.ab. or groups.ab.
22	184108	((singl$ or doubl$ or treb$ or tripl$) adj (blind$3 or mask$3)).tw
23	35723	(allocated adj2 random$).tw
24	7745771	Or/18-23
25	2690	17 and 24
26	1738	limit 25 to (english language and yr="2015 - 2021")

Cochrane Central Register of Controlled Trials (CENTRAL) from 1946 to November 11, 2021

#No.	Results	Searches
1	484	MeSH descriptor: [Endovascular Procedures] this term only
2	672	MeSH descriptor: [Vascular Surgical Procedures] this term only
3	327	MeSH descriptor: [Thrombectomy] explode all trees
4	11	MeSH descriptor: [Embolectomy] explode all trees
5	4394	MeSH descriptor: [Stents] explode all trees
6	18003	(stent* or thromboaspirat* or thrombo-aspirat* or thrombecto* or embolecto*):ti,ab,kw
7	928	((clot or thrombus or thrombi or embol*) NEAR/5 (aspirat* or remov* or retriev* or retract* or extract*)):ti,ab,kw
8	8415	((mechanical or endovascular or neurovascular) NEAR/5 (aspirat* or treatment* or therap*)):ti,ab,kw
9	68	((merci or concentric) NEAR/5 retriever):ti,ab,kw
10	26691	#1 or #2 or #3 or #4 or #5 or #6 or #7 or #8 or #9
11	1453	MeSH descriptor: [Cerebrovascular Disorders] this term only
12	10	MeSH descriptor: [Basal Ganglia Cerebrovascular Disease] this term only
13	3820	MeSH descriptor: [Brain Ischemia] explode all trees
14	491	MeSH descriptor: [Carotid Artery Diseases] this term only
15	18	MeSH descriptor: [Carotid Artery Thrombosis] this term only
16	12	MeSH descriptor: [Intracranial Arterial Diseases] this term only
17	26	MeSH descriptor: [Cerebral Arterial Diseases] this term only
18	324	MeSH descriptor: [Intracranial Embolism and Thrombosis] explode all trees
19	10731	MeSH descriptor: [Stroke] explode all trees
20	17215	(isch?emi* NEAR/5 (stroke* or apoplex* or cerebral vasc* or cerebrovasc* or cva or attack*)):ti,ab,kw
21	18229	((brain or cerebr* or cerebell* or vertebrobasil* or hemispher* or intracran* or intracerebral or infratentorial or supratentorial or middle cerebr* or mca* or anterior circulation) NEAR/5 (isch?emi* or infarct* or thrombo* or emboli* or occlus* or hypoxi*)):ti,ab,kw
22	34408	#11 or #12 or #13 or #14 or #15 or #16 or #17 or #18 or #19 or #20 or #21
23	3414	#10 and #22
24	2071	MeSH descriptor: [Intracranial Hemorrhages] explode all trees
25	19	MeSH descriptor: [Basal Ganglia Hemorrhage] explode all trees
26	11521	((brain* or cerebr* or cerebell* or intracerebral or intracrani* or parenchymal or intraparenchymal or intraventricular or infratentorial or supratentorial or basal gangli* or putaminal or putamen or posterior fossa or hemispher*)

		NEAR/5 (h?emorrhag* or h?ematoma* or bleed*)):ti,ab,kw
27	1351	((h$emorrhag* or bleed*) NEAR/5 (stroke or apoplex*)):ti,ab,kw
28	2788	(ICH or ICHs):ti,ab,kw
29	14201	#24 or #25 or #26 or #27 or #28
30	895	#23 and #29
31	682	#23 with Publication Year from 2015 to 2021, in Trials

Embase from 1947 to November 11, 2021

#No.	Results	Searches
1	275,431	'endovascular surgery'/de OR 'stent'/exp OR 'vascular surgery'/de OR 'thrombectomy'/exp OR 'embolectomy'/exp OR 'thrombectomy device'/exp
2	208,367	(stent* OR thromboaspirat* OR 'thrombo aspirat*' OR thrombecto* OR embolecto*):ti,ab,kw
3	11,978	((clot OR thrombus OR thrombi OR embol*) NEAR/5 (aspirat* OR remov* OR retriev* OR retract* OR extract*)):ti,ab,kw
4	53,355	((mechanical OR endovascular OR neurovascular) NEAR/5 (aspirat* OR treatment* OR therap*)):ti,ab,kw
5	196	((merci OR concentric) NEAR/5 retriever):ti,ab,kw
6	347,937	#1 OR #2 OR #3 OR #4 OR #5
7	565,209	'cerebrovascular disease'/de OR 'basal ganglion hemorrhage'/de OR 'brain ischemia'/exp OR 'carotid artery disease'/de OR 'cerebral artery disease'/de OR 'arterial thromboembolism'/exp OR 'cerebrovascular accident'/de OR 'ischemic stroke'/exp
8	142,316	(isch$emi* NEAR/5 (stroke* OR apoplex* OR 'cerebral vasc*' OR cerebrovasc* OR cva OR attack*)):ti,ab,kw
9	130,520	((brain OR cerebr* OR cerebell* OR vertebrobasil* OR hemispher* OR intracran* OR intracerebral OR infratentorial OR supratentorial OR 'middle cerebr*' OR mca* OR 'anterior circulation') NEAR/5 (isch?emi* OR infarct* OR thrombo* OR emboli* OR occlus* OR hypoxi*)):ti,ab,kw
10	634,330	#7 OR #8 OR #9
11	50,505	#6 AND #10
12	161,917	'basal ganglion hemorrhage'/exp OR 'brain hemorrhage'/exp
13	103,197	((brain* or cerebr* or cerebell* or intracerebral or intracrani* or parenchymal or intraparenchymal or intraventricular or infratentorial or supratentorial or 'basal gangli*' or putaminal or putamen or 'posterior fossa' or hemispher*) NEAR/5 (h$emorrhag* or h$ematoma* or bleed*)):ti,ab,kw
14	28,540	((h$emorrhag* or bleed*) NEAR/5 (stroke or apoplex*)):ti,ab,kw
15	27,890	(ICH or ICHs):ti,ab,kw
16	212,877	#12 OR #13 OR #14 OR #15
17	11,379	#11 AND #16
18	11,094,918	'clinical study'/exp OR 'case control study'/exp OR 'family study'/exp OR 'longitudinal study'/exp OR 'retrospective study'/exp OR 'cohort analysis'/exp
19	716,655	'prospective study'/exp NOT 'randomized controlled trial (topic)'/exp
20	454,643	(cohort NEAR/5 (study OR studies)):ti,ab,kw

21	163,500	('case control' NEAR/5 (study OR studies)):ti,ab,kw
22	149,099	('follow up' NEAR/5 (study OR studies)):ti,ab,kw
23	280,518	(observational NEAR/5 (study OR studies)):ti,ab,kw
24	149,099	(epidemiologic* NEAR/5 (study OR studies)):ti,ab,kw
25	280,518	('cross sectional' NEAR/5 (study OR studies)):ti,ab,kw
26	1,949,470	'randomized controlled trial'/de OR 'clinical trial'/de OR 'controlled clinical trial'/de OR 'randomization'/exp OR 'placebo'/de OR 'control group'/de OR 'double blind procedure'/de OR 'single blind procedure'/de OR 'multicenter study'/de
27	4,096,506	('randomi$ed controlled trial*' OR 'controlled clinical trial*' OR 'clinical trial*' OR 'multicenter study' OR rct or placebo* OR 'drug therapy'):ti,ab,kw OR groups:ab
28	257,676	((singl* OR doubl* OR treb* OR tripl*) NEAR blind*):ti,ab,kw
29	48,201	(random* NEAR/2 allocat*):ti,ab,kw
30	13,576,973	OR/18-29
27	8,795	#17 AND #30
31	5,584	#27 AND [english]/lim AND [2015-2021]/py

Supplementary File 2 Newcastle-Ottawa Scale

NEWCASTLE - OTTAWA QUALITY ASSESSMENT SCALE
CASE CONTROL STUDIES

Note: A study can be awarded a maximum of one star for each numbered item within the Selection and Exposure categories. A maximum of two stars can be given for Comparability.

Selection

1) Is the case definition adequate?
   a) yes, with independent validation ✵
   b) yes, eg record linkage or based on self reports
   c) no description

2) Representativeness of the cases
   a) consecutive or obviously representative series of cases ✵
   b) potential for selection biases or not stated

3) Selection of Controls
   a) community controls ✵
   b) hospital controls
   c) no description

4) Definition of Controls
   a) no history of disease (endpoint) ✵
   b) no description of source

Comparability

1) Comparability of cases and controls on the basis of the design or analysis
   a) study controls for ________________ (Select the most important factor.) ✵
   b) study controls for any additional factor ✵ (This criteria could be modified to indicate specific control for a second important factor.)

Exposure

1) Ascertainment of exposure
   a) secure record (eg surgical records) ✵
   b) structured interview where blind to case/control status ✵
   c) interview not blinded to case/control status
   d) written self report or medical record only
   e) no description

2) Same method of ascertainment for cases and controls
   a) yes ✵
   b) no

3) Non-Response rate
   a) same rate for both groups ✵
   b) non respondents described
   c) rate different and no designation

NEWCASTLE - OTTAWA QUALITY ASSESSMENT SCALE
COHORT STUDIES

Note: A study can be awarded a maximum of one star for each numbered item within the Selection and Outcome categories. A maximum of two stars can be given for Comparability

Selection

1) Representativeness of the exposed cohort
 a) truly representative of the average _______________ (describe) in the community ✵
 b) somewhat representative of the average ______________ in the community ✵
 c) selected group of users eg nurses, volunteers
 d) no description of the derivation of the cohort

2) Selection of the non exposed cohort
 a) drawn from the same community as the exposed cohort ✵
 b) drawn from a different source
 c) no description of the derivation of the non exposed cohort

3) Ascertainment of exposure
 a) secure record (eg surgical records) ✵
 b) structured interview ✵
 c) written self report
 d) no description

4) Demonstration that outcome of interest was not present at start of study
 a) yes ✵
 b) no

Comparability

1) Comparability of cohorts on the basis of the design or analysis
 a) study controls for _____________ (select the most important factor) ✵
 b) study controls for any additional factor ✵ (This criteria could be modified to indicate specific control for a second important factor.)

Outcome

1) Assessment of outcome
 a) independent blind assessment ✵
 b) record linkage ✵
 c) self report
 d) no description

2) Was follow-up long enough for outcomes to occur
 a) yes (select an adequate follow up period for outcome of interest) ✵
 b) no

3) Adequacy of follow up of cohorts
 a) complete follow up - all subjects accounted for ✵
 b) subjects lost to follow up unlikely to introduce bias - small number lost - > ____ % (select an adequate %) follow up, or description provided of those lost) ✵
 c) follow up rate < ____% (select an adequate %) and no description of those lost
 d) no statement

Note: 1 ✵ means 1 point, and studies with scores of 0–4 points were identified as low quality and 5–9 points as high quality and only high-quality literature will be in our analysis.

Supplementary File 3 PSD$_{mRS}$ score 3-6 group's funnel plot favorable outcome

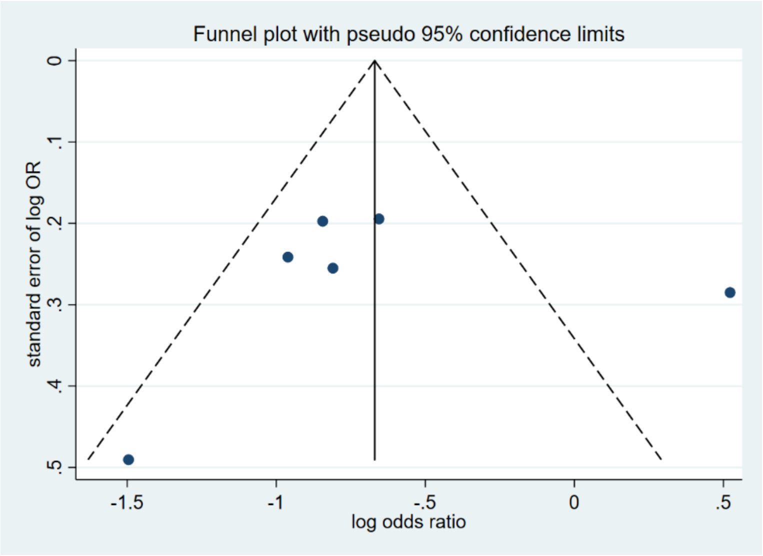

Supplementary File 4 PSD$_{mRS}$ score 3-6 group's funnel plot successful recanalization

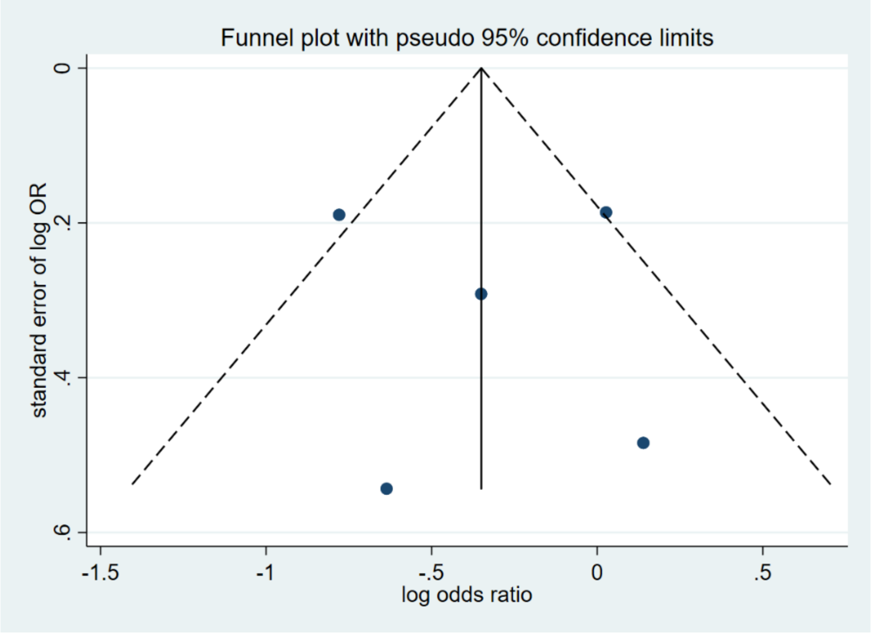

Supplementary File 5 PSD$_{mRS}$ score 3-6 group's funnel plot sICH

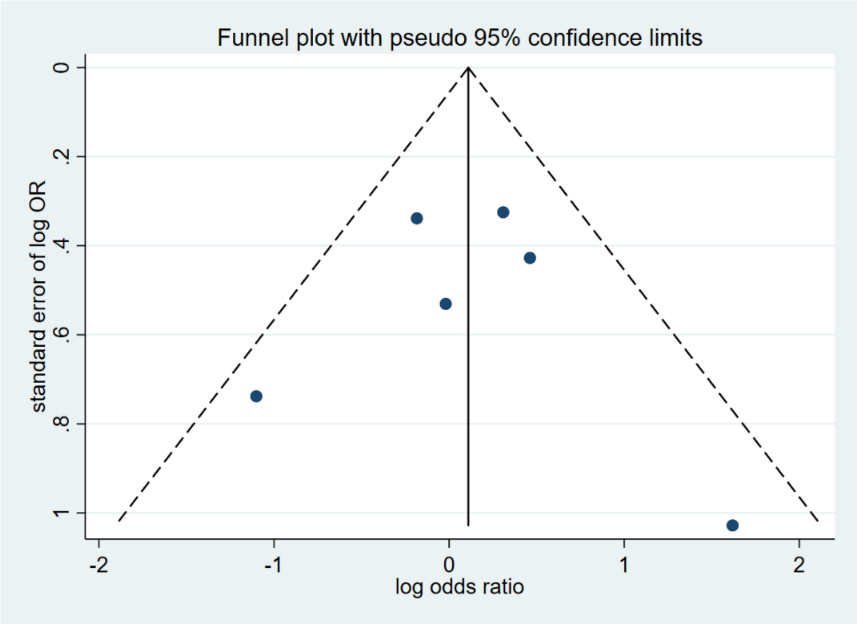

Supplementary File 6 PSD$_{mRS}$ score 3-6 group's funnel plot 90-day mortality

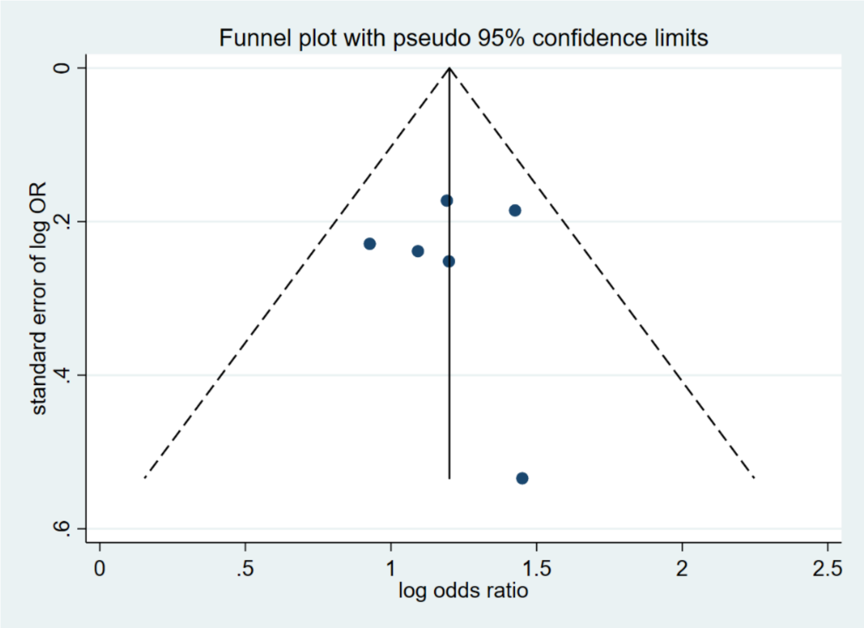

Supplementary File 7 PSD_{mRS} score 3-6 group's egger test favorable outcome

Egger's test for small-study effects:
Regress standard normal deviate of intervention
effect estimate against its standard error

.
Number of studies = 6 Root MSE = 2.39

Std_Eff	Coef.	Std. Err.	t	P>|t|	[95% Conf.	Interval]
slope	-.6300753	.9342933	-0.67	0.537	-3.224089	1.963939
bias	-.1682851	3.834498	-0.04	0.967	-10.81456	10.47799

Test of H0: no small-study effects P = 0.967

Supplementary File 8 PSD_{mRS} score 3-6 group's egger test successful recanalization

Egger's test for small-study effects:
Regress standard normal deviate of intervention
effect estimate against its standard error

.
Number of studies = 5 Root MSE = 1.865

Std_Eff	Coef.	Std. Err.	t	P>|t|	[95% Conf.	Interval]
slope	-.4249072	.5522883	-0.77	0.498	-2.182535	1.332721
bias	.3163246	2.153354	0.15	0.893	-6.536608	7.169257

Test of H0: no small-study effects P = 0.893

Supplementary File 9 PSD_{mRS} score 3-6 group's egger test sICH

Egger's test for small-study effects:
Regress standard normal deviate of intervention
effect estimate against its standard error

.
Number of studies = 6 Root MSE = 1.293

Std_Eff	Coef.	Std. Err.	t	P>|t|	[95% Conf.	Interval]
slope	.019963	.6797222	0.03	0.978	-1.867248	1.907174
bias	.2134216	1.520162	0.14	0.895	-4.007225	4.434069

Test of H0: no small-study effects P = 0.895

Supplementary File 10 PSD_{mRS} score 3-6 group's egger test 90-day mortality

```
Egger's test for small-study effects:
Regress standard normal deviate of intervention
  effect estimate against its standard error

.
Number of studies =   6                              Root MSE      =   .9129
------------------------------------------------------------------------------
     Std_Eff |      Coef.   Std. Err.      t    P>|t|     [95% Conf. Interval]
-------------+----------------------------------------------------------------
       slope |   1.209627     .29718     4.07   0.015     .3845226    2.034731
        bias |  -.041961    1.321313    -0.03   0.976    -3.710514    3.626592
------------------------------------------------------------------------------

Test of H0: no small-study effects          P = 0.976
```

Supplementary File 11 PSD_{mRS} score 3-6 group's funnel plot adjusted favorable outcome

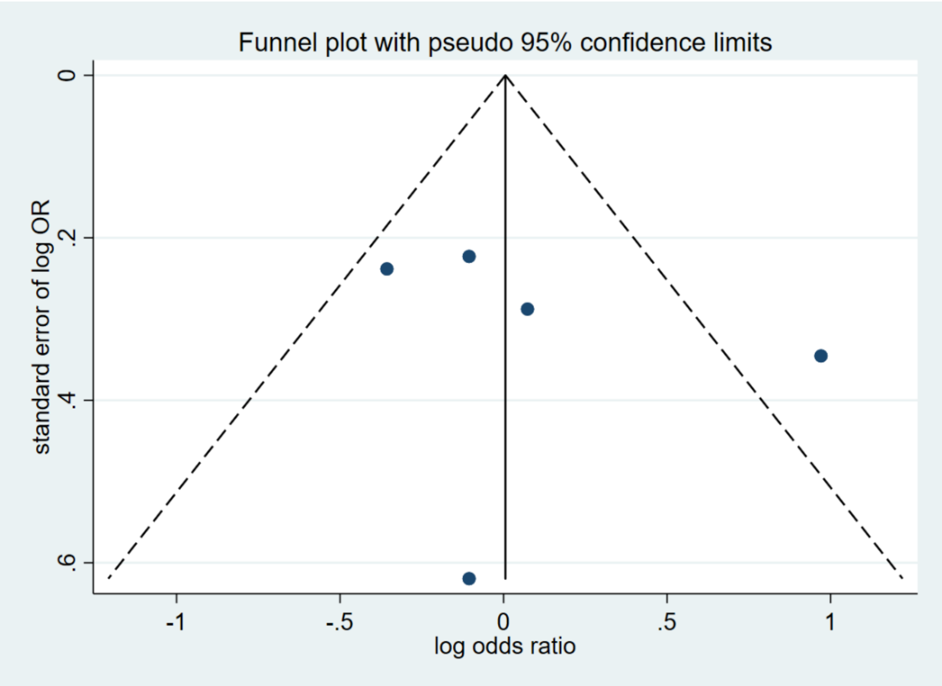

Supplementary File 12 PSD_{mRS} score 3-6 group's funnel plot adjusted sICH

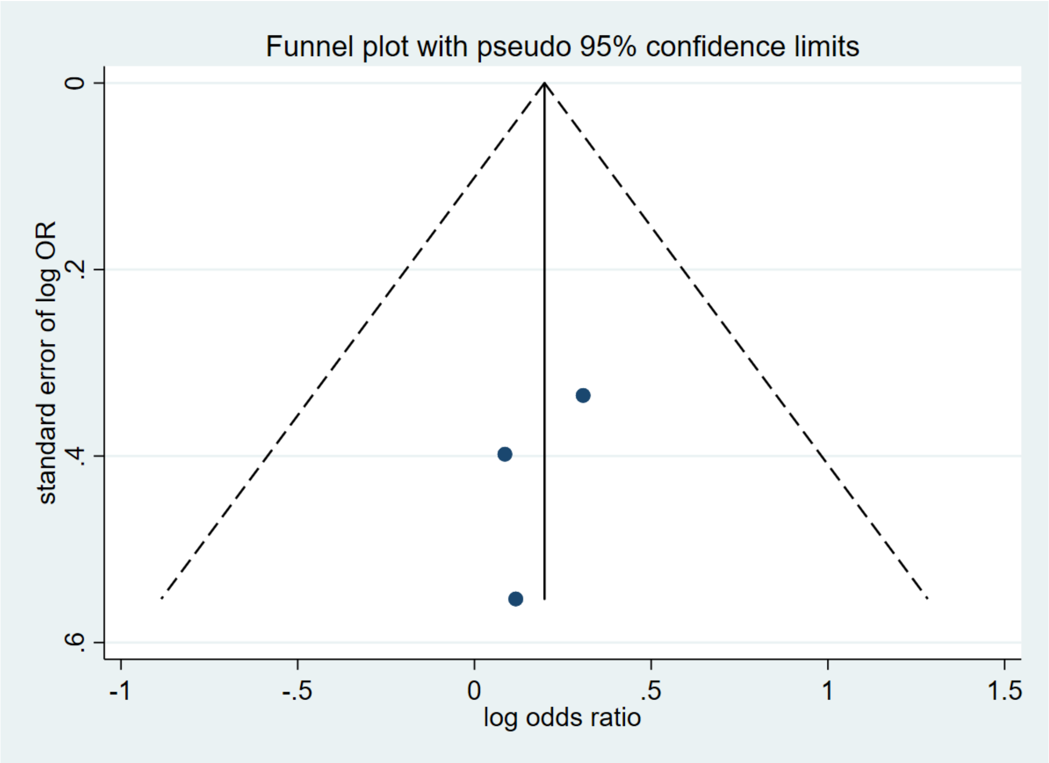

Supplementary File 13 PSD_{mRS} score 3-6 group's funnel plot adjusted 90-days mortality

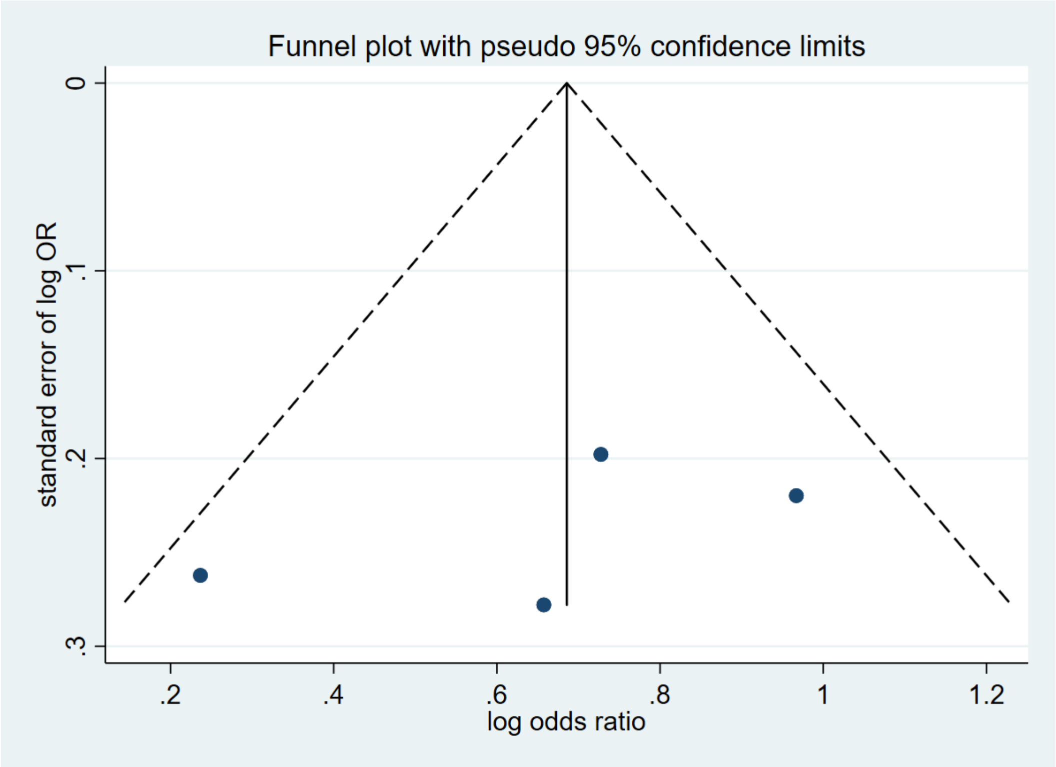

Supplementary File 14 PSD_{mRS} score 3-6 group's egger test adjusted favorable outcome

Number of studies = 5 Root MSE = 1.718

Std_Eff	Coef.	Std. Err.	t	P>|t|	[95% Conf.	Interval]
slope	-.5226555	.7505621	-0.70	0.536	-2.911279	1.865968
bias	1.927116	2.61683	0.74	0.515	-6.400804	10.25504

Test of H0: no small-study effects P = 0.515

Supplementary File 15 PSD_{mRS} score 3-6 group's egger test adjusted sICH

Egger's test for small-study effects:
Regress standard normal deviate of intervention
effect estimate against its standard error

Number of studies = 3 Root MSE = .3389

Std_Eff	Coef.	Std. Err.	t	P>|t|	[95% Conf.	Interval]
slope	.5539669	.4044561	1.37	0.401	-4.585135	5.693068
bias	-.9003393	1.004078	-0.90	0.535	-13.65836	11.85769

Test of H0: no small-study effects P = 0.535

Supplementary File 16 PSD_{mRS} score 3-6 group's egger test adjusted 90-day mortality

Egger's test for small-study effects:
Regress standard normal deviate of intervention
effect estimate against its standard error

Number of studies = 4 Root MSE = 1.268

Std_Eff	Coef.	Std. Err.	t	P>|t|	[95% Conf.	Interval]
slope	1.696238	1.089042	1.56	0.260	-2.989531	6.382006
bias	-4.379424	4.675976	-0.94	0.448	-24.49852	15.73968

Test of H0: no small-study effects P = 0.448

Supplementary File 17 PSD_{mRS} score 2-6 group's funnel plot favorable outcome

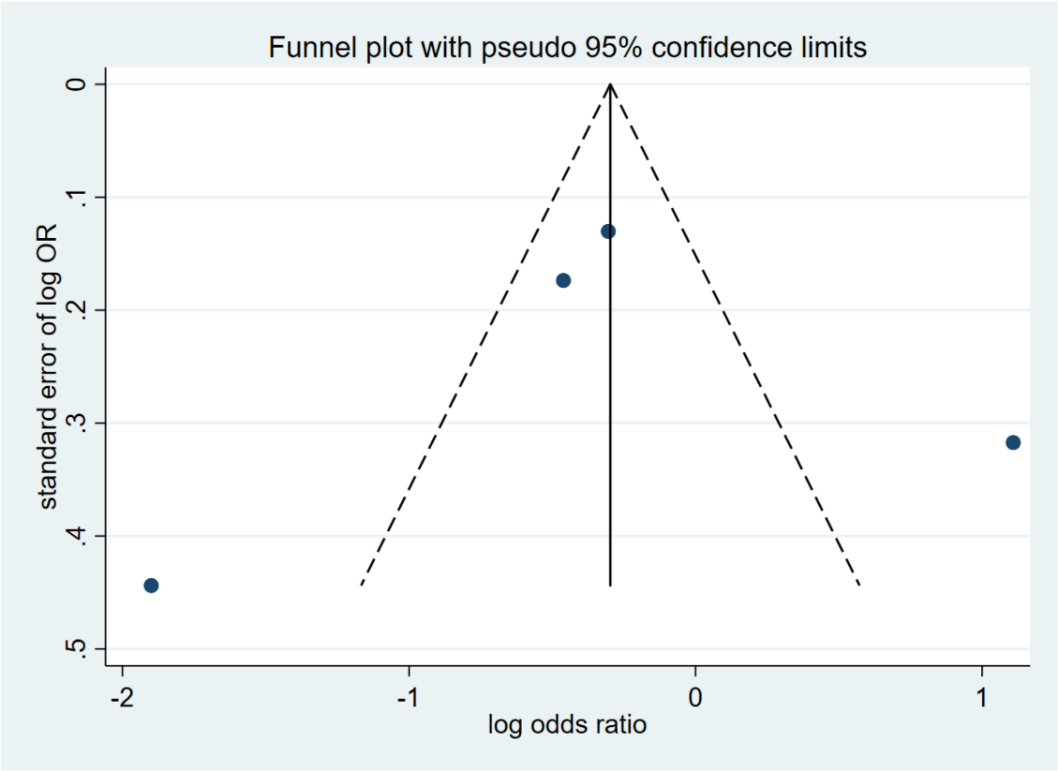

Supplementary File 18 PSD_{mRS} score 2-6 group's funnel plot 90-day mortality

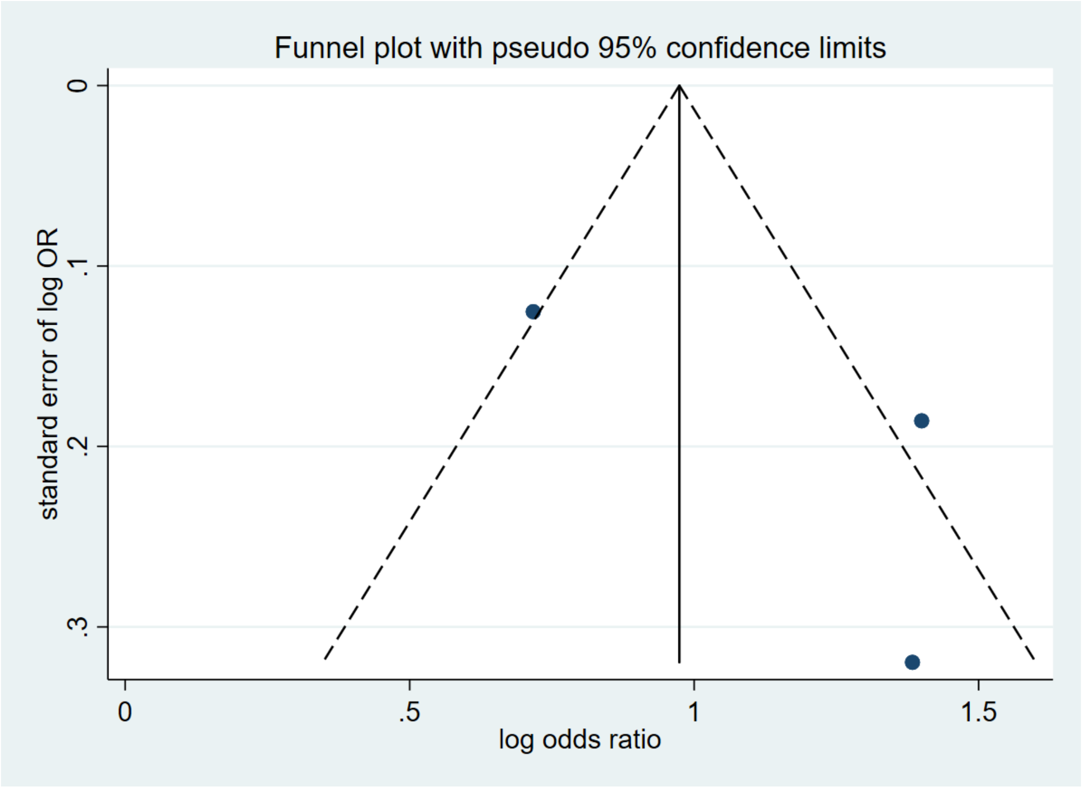

Supplementary File 19 PSD$_{mRS}$ score 2-6 group's egger test favorable outcome

```
Egger's test for small-study effects:
Regress standard normal deviate of intervention
  effect estimate against its standard error

.
Number of studies =   4                                    Root MSE       =    4.094
```

Std_Eff	Coef.	Std. Err.	t	P>|t|	[95% Conf.	Interval]
slope	-.2529672	.9551806	-0.26	0.816	-4.362777	3.856843
bias	-.2528459	4.942525	-0.05	0.964	-21.51881	21.01312

```
Test of H0: no small-study effects          P = 0.964
```

Supplementary File 20 PSD$_{mRS}$ score 2-6 group's egger test 90-day mortality

```
Egger's test for small-study effects:
Regress standard normal deviate of intervention
  effect estimate against its standard error

.
Number of studies =   3                                    Root MSE       =    2.087
```

Std_Eff	Coef.	Std. Err.	t	P>|t|	[95% Conf.	Interval]
slope	.2626277	.6077991	0.43	0.740	-7.460193	7.985448
bias	4.419479	3.551975	1.24	0.431	-40.71265	49.55161

```
Test of H0: no small-study effects          P = 0.431
```

Supplementary File 21 PSD$_{mRS}$ score 2-6 group's funnel plot adjusted favorable outcome

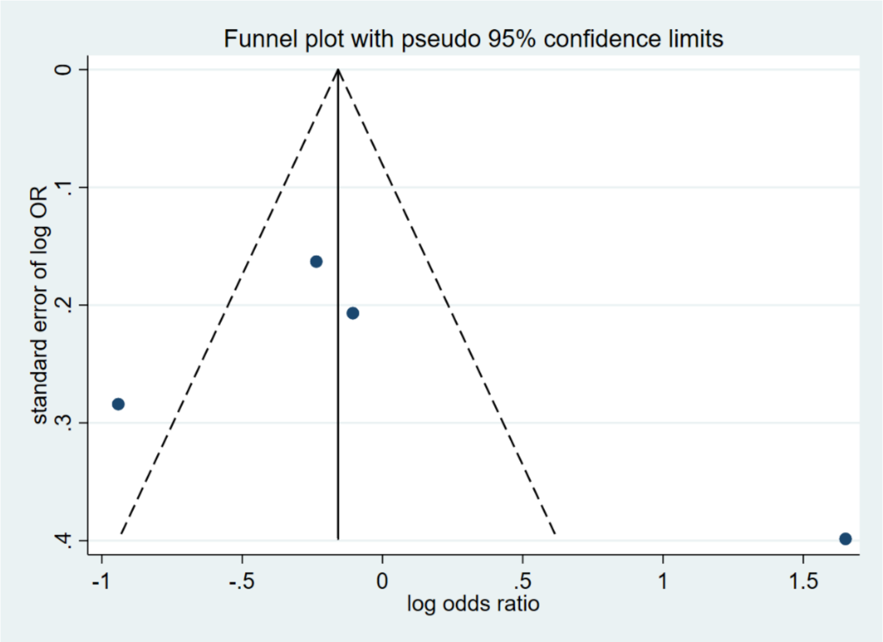

Supplementary File 22 PSD$_{mRS}$ score 2-6 group's funnel plot adjusted 90-days mortality

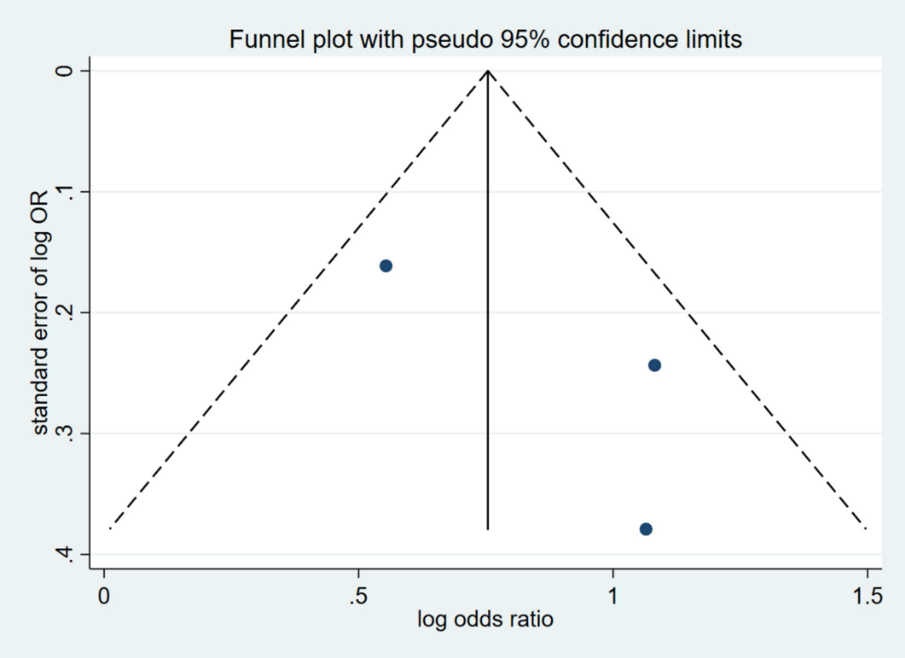

Supplementary File 23 PSD$_{mRS}$ score 2-6 group's egger test adjusted favorable outcome

.
Number of studies = 4 Root MSE = 3.315

Std_Eff	Coef.	Std. Err.	t	P>|t|	[95% Conf.	Interval]
slope	-1.048139	1.214291	-0.86	0.479	-6.27281	4.176532
bias	4.171718	5.41948	0.77	0.522	-19.14642	27.48986

Test of H0: no small-study effects P = 0.522

Supplementary File 24 PSD$_{mRS}$ score 2-6 group's egger test adjusted 90-day mortality

.
Number of studies = 3 Root MSE = 1.118

Std_Eff	Coef.	Std. Err.	t	P>|t|	[95% Conf.	Interval]
slope	.1309239	.4414316	0.30	0.816	-5.477996	5.739844
bias	2.996551	2.010894	1.49	0.376	-22.55427	28.54738

Test of H0: no small-study effects P = 0.376
